# Of mitogens and morphogens: modelling Sonic Hedgehog mechanisms in vertebrate development

**DOI:** 10.1098/rstb.2019.0660

**Published:** 2020-08-24

**Authors:** Ian Groves, Marysia Placzek, Alexander G. Fletcher

**Affiliations:** 1 School of Mathematics and Statistics, University of Sheffield, Hicks Building, Hounsfield Road, Sheffield S3 7RH, UK; 2 Department of Biomedical Science, University of Sheffield, Firth Court, Western Bank, Sheffield, S10 2TN, UK; 3 Bateson Centre, University of Sheffield, Firth Court, Western Bank, Sheffield, S10 2TN, UK

**Keywords:** Sonic Hedgehog, patterning, growth, morphogenesis, developmental biology, mathematical modelling

## Abstract

Sonic Hedgehog (Shh) Is a critical protein in vertebrate development, orchestrating patterning and growth in many developing systems. First described as a classic morphogen that patterns tissues through a spatial concentration gradient, subsequent studies have revealed a more complex mechanism, in which Shh can also regulate proliferation and differentiation. While the mechanism of action of Shh as a morphogen is well understood, it remains less clear how Shh might integrate patterning, proliferation and differentiation in a given tissue, to ultimately direct its morphogenesis. In tandem with experimental studies, mathematical modelling can help gain mechanistic insights into these processes and bridge the gap between Shh-regulated patterning and growth, by integrating these processes into a common theoretical framework. Here, we briefly review the roles of Shh in vertebrate development, focusing on its functions as a morphogen, mitogen and regulator of differentiation. We then discuss the contributions that modelling has made to our understanding of the action of Shh and highlight current challenges in using mathematical models in a quantitative and predictive way.

This article is part of a discussion meeting issue ‘Contemporary morphogenesis’.

## 1. Introduction

Sonic Hedgehog (Shh) is a secreted glycoprotein encoded by the *Shh* gene. First cloned in the 1990s, on the basis of its high conservation with the *Drosophila* *hedgehog* (*hh*) gene [1–6], Shh is the best studied ligand of the hedgehog family and plays a key role in vertebrate development and organogenesis. Initial studies (described below) revealed the importance of Shh in patterning the embryonic ventral neural tube and posterior limb bud. These studies were followed swiftly by those indicating that Shh plays an important role in directing a vast array of developmental processes in the embryo, including development of the somites [7], foregut [8], lung [9] and brain [10–12], as well as craniofacial development [13–16], and is instrumental in directing cell proliferation in particular embryonic populations [17]. Further investigations showed Shh has diverse functional roles in fetal and postnatal life, in circuit wiring and in stem cell regulation [18–22].

Here, we briefly summarize the current state of knowledge regarding the roles of Shh as a morphogen and a mitogen in the embryo, before focusing on the utility of mathematical modelling in dissecting the complexity of Shh activity. Through illustrative examples, we discuss how different modelling approaches have allowed mechanistic insights into Shh-controlled gene expression at the cellular level, as well as the actions of Shh as a mitogen and a morphogen at the tissue scale. We conclude by discussing how mathematical modelling could help future efforts to study the multifunctional nature of Shh signalling throughout development.

## 2. Sonic Hedgehog as a morphogen

Turing introduced the concept of a morphogen [23] and subsequent studies [24,25] led to its conventional definition: a molecule that diffuses through cells and tissues to establish a concentration gradient that evokes discrete cell responses at particular threshold concentrations to confer position identity and pattern cell/tissue fields. Classic grafting studies in the chick had suggested that the dorsoventral (DV) axis of the posterior neural tube (the future spinal cord) and anterior–posterior (AP) axis of the limb bud are patterned through the activity of a morphogen deriving from ventral midline cells of the notochord and floor plate [26], and the posterior zone of polarizing activity (ZPA) ([27], respectively. The cloning of Shh provided insight into the molecular identity of the morphogen: Shh showed restricted expression to the notochord, floor plate and ZPA [1–4,6].

**Figure 1.**
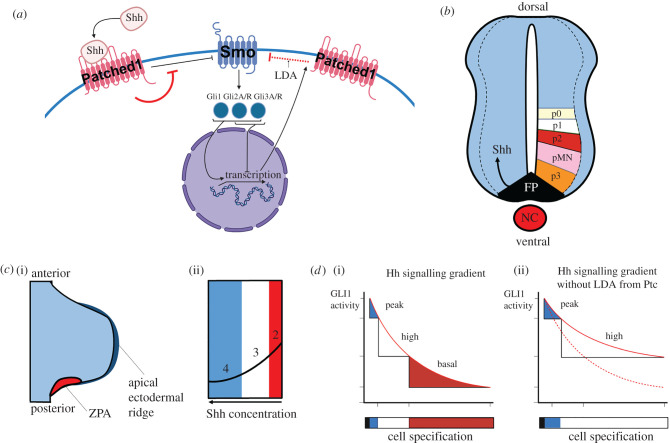
Key features of the Shh pathway. (*a*) Simplified schematic of the ‘canonical’ Shh pathway. Shh binds to the membrane-bound receptor Patched1, relieving Patched1's constitutive inhibition of Smoothened (Smo). Upon Shh signalling, Smo is thus able to interact with Gli transcription factors, which initiate transcription of Shh target genes such as the gene encoding Patched1. This gives rise to ligand-dependent antagonism (LDA; red dotted flathead arrow), whereby Shh network activity stimulates the expression of its own repressor (Ptc). Red flathead arrows indicate effects that occur in the presence of Shh binding. (*b*) Simplified schematic of neural tube patterning by Shh. Shh is expressed by the notochord (NC), and movement of Shh into the floor plate (FP) induces Shh expression in the FP. From here, Shh forms a concentration gradient from ventral to dorsal in the developing neural tube, specifying neural progenitors (p0–3, pMN). Dotted lines indicates progenitor region. (*c*) Simplified schematic of limb bud digit patterning by Shh. Shh is secreted from the zone of polarizing activity (ZPA) and travels through the posterior limb bud, specifying posterior identity through Gli1 and Gli3 induction. (*d*) Experimental evidence for Patched1-dependent ligand-dependent antagonism on developing hair follicles, adapted from [29]. (i) The levels of Gli1 activity resulting from a proximo-distal Shh concentration gradient. (ii) In a Patched1 genetic knockout, the Hedgehog gradient is no longer attenuated via ligand-dependent antagonism, so high concentration-dependent cell identities are found more distally. Red dotted line is the shape of the wild-type Shh gradient.

The canonical Shh signalling pathway involves effector zinc-finger transcription factors of the Gli family: Gli1, Gli2 and Gli3. Gli1 exists only as an activator, whereas Gli2 and Gli3 can be converted from repressor (GliR) to activator (GliA) forms (reviewed in [28]). The signalling pathway is initiated when secreted Shh binds Patched (Ptc) at the surface of a responsive cell. Binding relieves inhibition of the transmembrane protein Smoothened (Smo) and ultimately triggers the activation of the Gli transcription factors. This in turn results in activation of Shh target genes, including Ptc, forming a negative feedback loop (figure 1*a*).

Loss-of-function and gain-of-function studies of Shh, or components of its signalling pathway, indicated that Shh acts as a stereotypical morphogen in both the neural tube and the limb bud, i.e. establishing a concentration gradient that is translated into a GliA–GliR gradient that patterns these tissues and instruct cell fates (although note that the precise regulatory role of each Gli is not fully elucidated) [3,30]. Thus in the neural tube, Shh is secreted from the notochord and floor plate and diffuses through ventral regions of the neural tube, converting Gli2 and Gli3 to GliA forms, and inducing Gli1 (a GliA) [31]. In turn, this leads to the establishment of different progenitor cells types along the DV axis (figure 1*b*). In the developing limb bud, Shh is secreted from the ZPA, travels through the posterior limb bud and confers posterior identity through canonical induction of Gli1 and prevention of Gli3R [32] (figure 1*c*). Thus in both systems, the relative levels of GliA–GliR, and the balance between activation and repression of target genes, are the pivotal mechanism by which cells translate a gradient of Shh ligand into a discrete set of cell identities (reviewed in [31,33,34]). The mechanisms through which Shh becomes spatially distributed in both the neural tube and limb bud remain poorly understood (reviewed in [33,34]). Candidate mechanisms include diffusion that is free [24] or diffusion that is modified by lipid modification of Shh, or its binding to proteoglycans in the extracellular matrix [35].

Importantly, the timing of exposure of cells to Shh—in particular the length of their exposure—is critical to a cell's development: cells are capable of measuring their own exposure to Shh and integrating this as meaningful information over periods of time [36,37]. This challenges the conventional definition as a morphogen, as it means that absolute levels of Shh are not directly translated into a spatial ‘positional’ value [38]. This is perhaps most well characterized in the embryonic chick. Here, in both the neural tube and the limb bud, Shh-responsive cells integrate Shh levels over time and transiently progress through progenitor identities, promoting to sequential ventral, or posterior identities [39,40]. Furthermore, cells can become refractory to Shh over time, an event that is triggered by the Shh-induced negative feedback loop described above. Thus, the higher the amount of Shh signalling over time, the more the pathway is suppressed, a mechanism termed ligand-dependent antagonism (reviewed in [41]) (figure 1*d*). These dynamic responses indicate that to understand Shh's action as a morphogen it is critical to study its effects in both space and time. As we will discuss, such advanced mechanistic understanding greatly benefits from mathematical modelling.

## 3. Sonic Hedgehog functions as a mitogen and regulates the cell cycle

A less well-characterized role for Shh is as a mitogen (reviewed in [41]), a role that it orchestrates by altering cell cycle kinetics. First described as being critical for proliferation of granule neuron progenitors in the developing cerebellum [17,22,42], Shh signalling is now known to govern cell proliferation in many tissues. In the chick limb bud, classic studies suggested an integration of growth and patterning [43], and more recently Shh's influence on the cell cycle has been characterized: Shh signalling first stimulates ZPA cell proliferation via Cyclin D2 before downregulating proliferation in the ZPA through control of BMP2 signalling [44]. This fine control of proliferation by Shh ensures the correct number of digits form in the limb. In spinal cord progenitors, Shh signalling regulates the length of the G1 phase of the cell cycle, decreases cell cycle length and increases expression of *Cyclin D1* and *N-myc*, to expand specific progenitor pools [45,46]. In the brain, Shh induces progenitor cell proliferation and the maintenance or growth of progenitor cell populations [47]; similarly, Shh has a proliferative role in retinal, hypothalamic and telencephalic progenitors [48–50] (see reviews by [46,47]). The specific effectors regulating the mitogenic activity of Shh are likely to vary across tissues [51]: for example, the phosphatase Eya1 is known to lie upstream of Shh-controlled symmetrical cell divisions in the granule precursor cells [52]. Moreover, an important feature of Shh control of proliferation is timing, as Shh first promotes cell cycle progression but then inhibits it [37,44], and in this manner, can play a role in cell cycle exit.

Indeed, in many systems, Shh also governs cell differentiation (reviewed in [53]). Studies suggest that this can occur as Shh triggers the transcription of signalling pathways that feedback to promote cell cycle arrest, or to antagonize Shh signalling [54]. In the hypothalamus, Shh may ultimately upregulate p57, driving cell cycle exit [49]; in the developing thymus gland, auto-repression of Shh signalling by Gli3 stimulates differentiation [55]. It remains unclear whether these events are context-dependent or are a common mechanism of differentiation regulation by Shh. Other cell behaviours, directly governed by Shh, may likewise govern cell differentiation. Increasing numbers of studies reveal that Shh can control the plane of cell division [52], as well as cell orientation and migration [56,57], each of which could intrinsically direct an exit from the cell cycle.

In summary, our understanding of the action of Shh has undergone a dramatic change in recent decades. Shh does not simply provide positional information by establishing a spatial morphogenic field, but instead triggers complex downstream effects that control the entire process of morphogenesis: patterning, proliferation, growth and differentiation. The open questions that remain, however, are: how are patterning, proliferation, growth and differentiation integrated, and what properties do their integration confer that we cannot understand by taking each alone?

Traditionally, the actions of Shh have been dissected through genetic or pharmacological interventions, conducted and analysed at specific time-points. While these approaches have enormous merit and are highly tractable, consideration needs to be given to the idea that such studies will inevitably miss many dynamic events, given that Shh is operating under tight timescales and has strong positive and negative feedback loops. Further, it is simply not feasible to conduct interventions at repeated time-points during development. As will be discussed below, a potential solution to this issue is the analysis of Shh in development via mathematical modelling, an approach that lends itself to the analysis of the multifunctional Shh pathway in a systematic, quantitative and predictive manner.

## 4. A role for mathematical modelling

Mathematical modelling refers to the use of mathematical language to describe the behaviour of a system and is becoming an increasingly important component of the developmental biologist's ‘toolbox’ [58,59]. Mathematics has several uses as a tool for understanding complex scientific phenomena. First, mathematics enables formalization: early biological examples include Fisher's interpretation of Darwin's theory of natural selection [60] and Turing's general mechanism of pattern formation through diffusion-driven instability [23]. Second, the precision of mathematics allows us to obtain a quantitative and predictive understanding of specific phenomena: an early biological example is Hodgkin & Huxley's modelling of action potentials in neurons and cardiomyocytes [61]. The scale at which developmental processes can be modelled has undergone a step-change in recent years, as large-scale data collection methodologies, advances in genetics, manipulation of cellular behaviours and light microscopy are allowing quantitative descriptions and analyses down to the nanoscale [62].

When constructing a mathematical model of a biological system, it is usual to adopt a ‘modular’ approach, where the component parts of the model are chosen on the basis of existing experimental data and the questions being asked of the system [63]. Such an approach may be compared with the experimental investigation of developmental processes through modular perturbations such as genetic knockouts. Thus, a useful starting point is to construct a simple or generic model that neglects fine-grained details but helps improve our qualitative understanding of the mechanisms that can give rise to important features of development such as robustness [64]. We can then refine a model in the light of new experimental data and increase the level of complexity, such as including molecular details or spatial effects. For the remainder of the review, we will discuss how mathematical approaches have given insights into the actions of Shh, broadly following this concept of increasing complexity; for a more detailed discussion of the underlying mathematics, see e.g. [65].

## 5. Modelling Sonic Hedgehog-controlled gene expression

**Figure 2.**
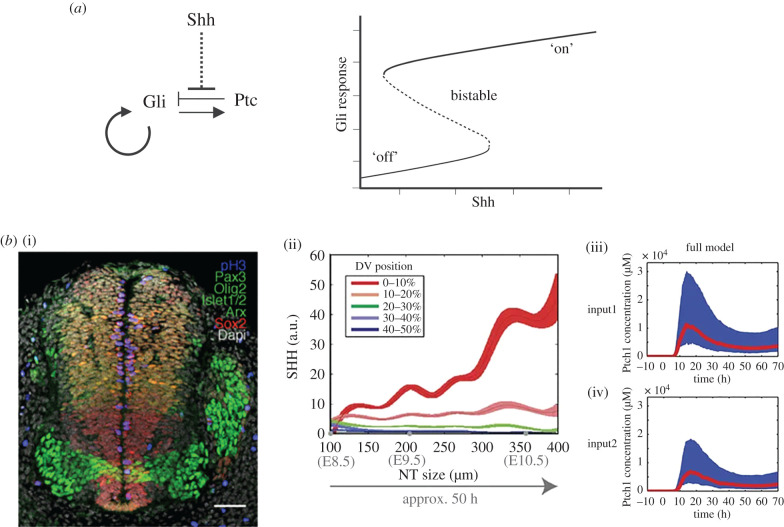
Modelling Shh-controlled gene expression. (*a*) (i) Key feedbacks present in the model in [66]. (ii) Bifurcation analysis of the model in [66]. The model exhibits bistability, demonstrating that a graded input of Shh can result in a binary response. (*b*) (i) Example of transverse section of the developing neural tube, molecularly labelled for genes that indicate DV identity, from [67]. (ii) Experimental measurements of Shh levels from [68]. (iii, iv) Fitting of the data to the mathematical model in [68], with high (upper) and low (lower) expression of Shh. a.u., arbitrary units.

A major focus of mathematical modelling to date has been to understand how Shh effects changes in signal transduction and gene regulation. Lai *et al.* [66] proposed the first mathematical model of the gene regulatory network downstream of Shh, focusing on how this network can switch fate choices at a threshold Shh concentration. Adopting the ‘start simple’ approach outlined above, the authors simplify the network by considering only the receptor Ptc and the transcription factors Gli1, Gli3 and Gli3R, and—to reduce complexity and because the activities of Gli1 and Gli2 are deemed by the authors to overlap—a lumped term ‘Gli’ representing the effects of both these factors (figure 2*a*). Their model comprises a set of coupled ordinary differential equations (ODEs), which describe how the concentrations of the network components change smoothly over time owing to Shh binding to Ptc, the basal and inducible activities of the *gli* and *ptc* promoters, and the constitutive degradation of each component.

By mathematically analysing the qualitative behaviour of this model, the authors find that the network's ability to function as a genetic switch is due to a tight combination of positive feedback (Gli upregulates its own expression) and negative feedback (Gli upregulates its repressor, Ptc) (figure 2*a*). In the language of systems biology, this behaviour is called a toggle switch [69] and is one of the common network motifs found in nature. Unlike irreversible switches underlying ‘points of no return’, such as apoptosis [70], the system can switch from the low Gli state to the high Gli state and back again, if the Shh concentration is increased and decreased enough. To explore whether fluctuations in Shh concentration could undermine the genetic switch, Lai *et al.* [66] used a stochastic modelling approach, which accounts for intrinsic noise in transcriptional processes. Through stochastic simulation of their model, the authors identified a role for the negative Ptc feedback loop in dampening Gli fluctuations, thereby reducing the likelihood for such back-and-forth switching to occur.

This work highlights how mathematical modelling can refine our mechanistic understanding of Shh-controlled gene expression. In addition, the model is capable of generating experimentally testable predictions. For example, parameter sensitivity analysis revealed that an increase in the maximal rate of inducible Gli transcription can lead to the genetic switch becoming irreversible: if the Shh concentration increases above a threshold, the system reaches and stays in a high Gli state, no matter how much the Shh concentration is later decreased. Such behaviour is predicted in cells with mutations resulting in constitutively active Smo.

While useful, the simplified and qualitative nature of the above model prevents more detailed predictions from being made. A more recent example that addressed these limitations is provided by Cohen *et al.* [68], who made use of approximate Bayesian computation (ABC) to inform a mathematical model of gene expression in the developing neural tube (figure 2*b*). This demonstrates how we can estimate the values of model parameters such as the rate that Shh binds Ptc, and quantify our uncertainty in these estimates, based on *in vivo* measurements. In other work, Cohen *et al.* [71] modelled the transcriptional network downstream of Shh. This model differs from [66], in that the investigation into the Shh-regulated transcriptional network is done in the biological context of the developing neural tube. Additionally, Cohen *et al.* fitted their model to biological data of gene expression domains, using wild-type and mutants to provide this basis. This model is more complex and attempts to incorporate more biological features of the Shh network. The network of downstream transcription factors analysed in this model are able, through their combinatorial activity, to produce a sharp output response around a neutral point; either side of this point the produced effect is opposite. Taken together the above two studies provide a useful framework to better understand how graded morphogenetic signals can produce sharp and distinct responses, adding to our understanding of how distinct progenitor domains are formed at different axial levels in developing tissues (figure 1*b*,*c*).

## 6. Modelling Sonic Hedgehog as a morphogen or mitogen

Another focus of modelling has been to understand how Shh acts as a morphogen within a developing tissue. As outlined in §2, the conventional definition of a morphogen is a molecule that provides positional information by inducing distinct cell types/cell signatures in a concentration-dependent manner. Perhaps the most well-known conceptual description of positional information is the ‘French flag model’, which refers to the autonomous formation of a spatial morphogen gradient within a tissue, with individual cells in the tissue interpreting the local concentration gradient to inform their fate. This elegant idea emerged through studies of the vertebrate limb and became a core concept in developmental biology [72].

**Figure 3.**
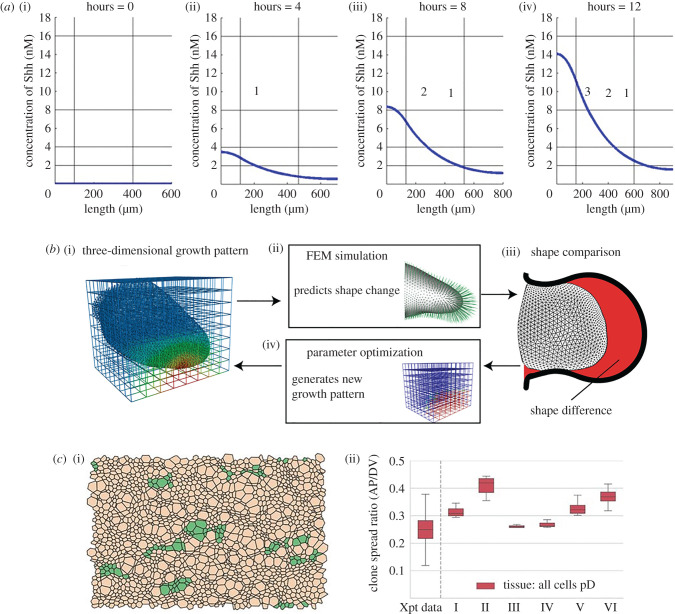
Modelling Shh in patterning and growth. (*a*) Simulation of chick wing digit specification by a Shh gradient in a uniformly linearly growing domain. Vertical black lines delineate the domain into three sections; horizontal black lines illustrate the thresholds required for each digit identity. Reproduced with permission from [73]. (*b*) Schematic illustrating how parameters in a three-dimensional model of the developing limb are fitted by comparing simulated shape dynamics to *in vivo* data. FEM, finite element model. Reproduced with permission from [74]. (*c*) Simulation of progenitor proliferation and differentiation in the developing neuroepithelium. (i) Simulated clones reveal a shape bias in the DV direction (dorsal progenitor cells are brown, green represents clones of these dorsal progenitor cells). (ii) Comparison of clone shape bias for six different parameter sets with *in vivo* data (Xpt data). pD, dorsal progenitor. Reproduced with permission from [75].

An example of how the mechanisms of Shh in development may be modelled mathematically is provided by Woolley *et al.* [73], who considered the Shh gradient formation and digit specification in a one-dimensional domain representing the chick wing bud. This domain was split into three distinct regions (posterior polarizing region where Shh is produced, digit-forming field and anterior region). The authors use a partial differential equation (PDE) approach to describe how the concentration of Shh evolves in time and space, owing to localized production, diffusion and decay of Shh within a uniformly growing domain. By prescribing a tissue growth rate the authors neglect any mitogenic activity of Shh, instead treating growth as an independent process which serves to dilute the Shh concentration. They found that, under the assumption that digit number is specified by the size of the digit-forming domain, this model could reproduce the temporal specification of the identities of the three chick wing digits (figure 3*a*). Furthermore, if digits were allowed to form from the polarizing region, this model could reproduce the four-digit pattern found in the chick leg. However, the model could not be extended in a straightforward manner to reproduce the five-digit pattern found in the mouse limb, suggesting that additional mechanisms must be present.

The study by Woolley *et al.* [73] illustrates how mathematical modelling can help to shed light on the maximal contribution of a specific mechanism to a developmental behaviour. In addition, the authors made modelling predictions that pave the way for future experimental validation and, in turn, model refinement. For example, the model assumes no movement of Shh outside of the limb bud and neglects known important components of the Shh network in the limb bud, such as the effects of BMP signalling (discussed in §3) [76] or intracellular signalling feedback events: these assumptions could be revised if needed, as informed by experimental evidence.

Alongside the French flag model, a second major theory for morphogen-directed pattern formation is that of diffusion-driven instability, whereby an initially homogeneous state breaks symmetry owing to the diffusion of, and reactions between, two chemical morphogens [23]. Originally developed in a general setting by Turing, subsequent studies made this theory more accessible by assigning specific characteristics to these morphogens: one being an ‘activator’ inducing positive feedback, the other being an ‘inhibitor’ inducing negative feedback [77]. Using such a model, Economou *et al.* [78] showed how development of the mammalian palate occurs through a Turing-type mechanism, with FGF10 as the activator and Shh as the inhibitor interacting to pattern the system. Subsequently, Menshykau *et al.* [79] also showed a similar mechanism occurred during the development of the embryonic lung. More recently, Menshykau *et al.* [80] demonstrated a similar potential role for Turing patterning in kidney morphogenesis. In contrast with Woolley *et al.* [73], this model requires the inclusion of the Shh receptor Ptc to successfully reproduce observed behaviour, in this case wild-type and mutant branching phenotypes.

Mathematical modelling has also been used to explore how a spatially and temporally evolving gradient of Shh signalling can be interpreted by the regulatory logic of the downstream transcriptional network. For example, in the context of neural tube development, Balaskas *et al*. [40] used an ODE model to interrogate and explain *in vivo* measurements and manipulations of the Shh network. Their model describes the temporal dynamics in expression of three neural tube genes downstream of Shh signalling (*Pax6, Olig2, Nkx2.2*), due to synthesis, degradation and cross-repressive interactions between these genes, and in response to Shh signalling. These three genes are used as a readout of the positional information the cell is receiving from Shh-induced Gli activity. Conducting a sensitivity analysis of the model, the authors found that with comparable degradation rates and repression levels, the behaviour of the system was robust. Low levels of Shh signalling coincided with increased Pax6 expression, moderate levels of Shh with increased Olig2 expression, and highest levels of Shh with high Nkx2.2 levels. The authors captured the presence of a Shh spatial gradient implicitly by analysing the model's response to different temporally varying Shh inputs. This work illustrates how mathematical modelling can help us to understand how morphogen interpretation emerges from the dynamical behaviour of complex transcriptional networks, rather than an intrinsic difference in individual gene responses to the morphogen.

While the above models neglect cell behaviours ‘downstream’ of Shh signalling, it is instructive to note the experimental demonstration by Xiong *et al.* [81] that active cell sorting of neural progenitors corrects any inconsistencies in Shh patterning to form sharp domains. This work indicates an uncoupling of cells' specification and their location and helps to explain how morphogen gradients can be scaled and operate over long distances. The mechanism described here confers robustness on the developing system, building upon the Shh signalling network that (e.g. through the modelling work by Lai *et al.* [66] described above) is known to be resilient to perturbation. We further discuss the inclusion of ‘downstream’ cell behaviours in mathematical models below.

Mathematical modelling has also been applied to help understand the role(s) of Shh as a mitogen. For example, Leffler *et al.* [82] developed a model to understand the action of Shh in the developing cerebellum [22,52,54]. This model consists of a set of coupled ODEs that describe how the numbers of proliferating granule cell precursors and differentiated granule cells change over time owing to symmetric division and differentiation of the former and exit of the latter from the external granule layer of the cerebellum. Their model showed interesting temporal features, such as a 2 day delay between highest number of proliferating granule progenitor cells in the outer and inner layers of the granule cell layer. This result predicts a regulatory mechanism *in vivo*—currently unknown—which is effecting this delay. Additionally Leffler *et al.* [82] ran simulations to examine growth dynamics in diseases, such as paediatric brain tumours, where too many granule cell precursors are generated. Thus, a promising avenue for future developmental biology and mathematical modelling studies is to examine and test our knowledge and assumptions about the relationship between patterning and growth in developmental disease.

## 7. Coupling growth and patterning in models of Sonic Hedgehog

A salient question, which is still not satisfactorily answered, is how patterning and growth couple to form coherent developmental structures. Considering that Shh functions as a morphogen and as a mitogen, it is thus a good candidate for answering this question mathematically. The models described in §6 focused on patterning and thus, for computational simplicity, tissue growth is an *imposed* rather than an *emergent* phenomenon in these models [73,80,83]: in each study, the authors prescribe tissue growth rates based on empirical measurements, instead of deriving equations for growth due to regulated cell proliferation, differentiation and death. An early model by Dillon & Othmer [84] including an explicit description of growth (i.e. so that growth is coupled to patterning, rather than being pre-determined) described the development of the vertebrate limb bud. Here, the growing tissue was described mathematically by a viscous fluid, whose volume increased over time owing to cell division. The rate of cell division was then assumed to be regulated by interactions between Shh and FGF signalling. The ability of the model to reproduce limb bud development was assessed through qualitative comparison of fluid particle trajectories to *in vivo* fate maps. A key insight of this work is that the explicit coupling of growth to patterning results in the predicted dynamics of a cell's exposure to the morphogens being much more heavily dependent on the cell's initial position in the early limb bud than would be predicted by a model with imposed growth.

While providing useful qualitative insights, the model by Dillon & Othmer [84] was proposed when there were limited quantitative data on growth dynamics. More recent work by Guerrero *et al.* [75] illustrates how such models can be placed on a quantitative footing. The authors develop and analyse a mechanical model of the developing neural tube, where every cell is represented by a polygon connected by vertices [85]. The neural tube shows anisotropic tissue growth: the DV axis grows significantly more than the anteroposterior axis (figure 3*c*). Through systematic comparison of their model using different parameter sets and mechanistic assumptions with *in vivo* measurements of tissue growth and cellular clone sizes and shapes, the authors found that such anisotropic growth can be attributed to a difference in differentiation rate between different progenitor domains specified by Shh signalling. This iterative process of building upon previous models to understand the modularity of development provides a blueprint for future growth-pattern-coupled models.

Further work has considered the role of growth in more complex three-dimensioal space in mathematical models. For example, Boehm *et al.* [74] used a computational model to examine whether differential cell proliferation rates were responsible for the shape changes seen in limb bud morphogenesis (figure 3*b*). As embryos change shape, the activity of morphogenetic signals can change, depending on the relative timescales of patterning and morphogenesis. In this first three-dimensional growth model of limb bud morphogenesis, the authors found, through parameter fitting, that purely proliferation was a *possible* explanation for limb bud outgrowth, but unlikely given the observed data and the acceptable parameter space in which the model could explain the data. This elegant study shows that there is a much greater complexity to morphogen-/mitogen-controlled development than a simple proliferation gradient. While this model did not explicitly account for Shh, a future incorporation to see how Shh would affect the results of the model would be interesting. This study represents a good example of an experiment–model–experiment cycle, as, after the authors discounted proliferation, they investigated whether oriented cell activities such as the axis of cell division were responsible for the outgrowth that is observed *in vivo*. Future work in this area could also include the effects of the ectoderm in shaping the developing limb bud.

Finally, Hiscock & Megason [86] discussed how in a Turing-type patterning system of activator and inhibitor there are three areas of control that determine the robust formation of patterns: gradients of activator/inhibitor (morphogen gradients), gradients of parameters (rates of differentiation) and tissue anisotropies (rates of growth). This work highlights the need to understand development at all these points of control and to try to integrate these three aspects into models attempting to further our understanding of morphogenesis.

## 8. Outlook

Shh has greatly informed our understanding of developmental processes, but also is a great case study for the construction of useful mathematical models of development. In particular, the roles of Shh as a morphogen and mitogen allow for mathematical models of both patterning and growth. As discussed above, these models have largely focused on *either* patterning *or* growth. Yet, in cases where Shh can act as a morphogen and mitogen, we require integrative models of patterning *and* growth.

One such example is the hypothalamus. The hypothalamus is of enormous importance, as it centrally regulates all core homeostatic mechanisms. These include sleep cycles, circadian rhythms and reproduction (reviewed in [87]). Since the year of its discovery, it has been known that Shh is involved in the development of the prospective hypothalamus [1]. Despite this, only recently has there been a clearer understanding of how Shh governs the development and growth of the hypothalamus. It has now been shown that, after acting as a morphogen to pattern the DV axis, Shh-expressing ventral hypothalamic progenitors produce progenitors that populate much of the basal hypothalamus through anisotropic growth [49] (reviewed in [88]). The key insight from this work is that hypothalamic progenitor cells concurrently specify as they grow and migrate anisotropically. This complexity is difficult to probe experimentally, owing to the narrow time windows and the complex regulatory networks involved. This remains a salient area of further research and a promising problem for modern techniques. A combined model of patterning and growth in the hypothalamus would need to account for the complex spatio-temporal dynamics of Shh and other key morphogens (Fgf10, BMP) in this tissue, as well as the dynamic and transient changes in tissue shape due to differential rates of proliferation/differentiation across the tissue. A key obstacle to calibrating such a model is the detailed experimental quantification of expression domains and characterization of developmental stages.

With regard to the future of mathematical models of Shh activity, there are promising avenues. For example, no model of Shh has included cell death in morphogenesis, let alone Shh-mediated cell death. This is relevant, as cell death has been suggested to play a key role in Shh-mediated developmental systems, such as the developing wing bud [89]. Additionally, new methods of obtaining and analysing biological data will make mathematical models more quantitative and provide opportunities to elucidate more subtle mechanisms and/or effects. Single cell RNA sequencing and super-resolution microscopy should provide the necessary basis on which to perform more detailed mathematical analyses and model calibration. Finally, with the advances made in modelling software and techniques, the field is now beginning to appreciate the importance of tissue geometry and shape in mathematical models of developing embryonic structures [90]. Further advances that focus on the role of cell/tissue shape in developing systems are likely to yield more exciting results.

We have reviewed the functions of Shh as a morphogen, a mitogen and a regulator of progenitor cell differentiation, focusing on the use of mathematical modelling to gain mechanistic insights. We conclude by highlighting the salient challenge in developmental biology: that of understanding how signalling ligands ultimately affect appropriate cellular behaviours and morphogenesis. The well-characterized activities of Shh, tools that enable exquisitely precise manipulation of its activity, and recent technological advances in light microscopy, live imaging, and tools to interpret, analyse, and model data, mean that Shh is an ideal candidate to investigate and overcome this challenge.

## Data accessibility

This article has no additional data.
